# Methicillin-Resistant and -Susceptible *Staphylococcus aureus* Infections in Dogs

**DOI:** 10.3201/eid1601.081758

**Published:** 2010-01

**Authors:** Meredith C. Faires, Michelle Traverse, Kathy C. Tater, David L. Pearl, J. Scott Weese

**Affiliations:** University of Guelph, Guelph, Ontario, Canada (M.C. Faires, D.L. Pearl, J.S. Weese); University of Pennsylvania, Philadelphia, Pennsylvania, USA (M. Traverse); Angell Animal Medical Center, Boston, Massachusetts, USA (K.C. Tater)

**Keywords:** Staphylococcus aureus, antimicrobial resistance, infection, risk factors, antimicrobial agents, fluoroquinolones, beta-lactams, dogs, bacteria, research

## Abstract

Methicillin-resistant *Staphylococcus aureus* (MRSA) has become a pathogen of animals. To compare types of infections, clinical outcomes, and risk factors associated with MRSA in dogs with those associated with methicillin-susceptible *Staphylococcus aureus* (MSSA) infections, we conducted a case–control study at 3 veterinary referral hospitals in the United States and Canada during 2001–2007. Risk factors analyzed were signalment, medical and surgical history, and infection site. Among 40 dogs with MRSA and 80 with MSSA infections, highest prevalence of both infections was found in skin and ears. Although most (92.3%) dogs with MRSA infections were discharged from the hospital, we found that significant risk factors for MRSA infection were receipt of antimicrobial drugs (odds ratio [OR] 3.84, p = 0.02), β-lactams (OR 3.58, p = 0.04), or fluoroquinolones (OR 5.34, p = 0.01), and intravenous catheterization (OR 3.72, p = 0.02). Prudent use of antimicrobial drugs in veterinary hospitals is advised.

## Abstract 2

Risk factors for MRSA include intravenous catheterization and receipt of certain antimicrobial drugs.

During the past 2 decades, methicillin-resistant *Staphylococcus aureus* (MRSA) has gained global attention as a human pathogen in hospitals and in communities. Recent reports of MRSA infection and colonization of dogs and cats (*1*–*5*) indicate that MRSA has apparently emerged as a pathogen of animals as well. Most reported MRSA infections in dogs have involved wound and postoperative infections (*2*), but evaluation is lacking regarding specific types of infections, clinical outcomes, and risk factors associated with such MRSA infections in dogs. No current evidence points to whether MRSA infections, in terms of location of infection, severity of disease, or clinical outcome, differ from methicillin-susceptible *S. aureus* (MSSA) infections.

The literature about human medicine has compared MRSA-infection risk factors (*6*,*7*), mortality rates (*6*,*8*–*10*), and clinical features (*6*) with those for MSSA infections. Results from a meta-analysis of 31 cohort studies showed that for patients with MRSA bacteremia, mortality rates were significantly higher than for patients with MSSA bacteremia (*11*). This mortality rate difference between MRSA and MSSA infections might result from treatment with inappropriate antimicrobial drugs or the restricted number of antimicrobial drugs available for treatment (*12*). With respect to animals, however, limited data are available; only 1 study has evaluated these MRSA and MSSA infections. Morris et al. (*13*) compared MRSA and MSSA infections in cats but were unable to detect significant differences in signalment, clinical presentations, or outcomes. Two other studies have reported potential risk factors associated with MRSA colonization in horses admitted to a veterinary referral hospital (*14*,*15*). Accurate data are needed to identify the differences between MRSA and MSSA infections in dogs as well as the clinical relevance of MRSA beyond concerns associated with antimicrobial drug resistance. To gain those data, epidemiologic research is required. Research is also required for proper medical treatment of dogs with MRSA infections, for counseling of clients of infected animals, and for elucidation of possible reasons for the emergence of MRSA in pets. Our study objectives were to compare the types of infections, clinical outcomes, and risk factors for MRSA and MSSA infections in dogs.

## Materials and Methods

### Selection of Case-Patients and Controls

From 2001 through 2007, we conducted a retrospective, secondary-base, case–control study at 3 veterinary referral hospitals: the Ontario Veterinary College Veterinary Teaching Hospital (Guelph, Ontario, Canada), Matthew J. Ryan Veterinary Hospital of the University of Pennsylvania (Philadelphia, PA, USA), and Angell Animal Medical Center (Boston, MA, USA). These hospitals offer a variety of small animal medical specialties and services. Each year they receive ≈14,000, 30,000, and 50,000 patients, respectively. Each hospital used its microbiology database to identify MRSA and MSSA infections in dogs. Each dog with an identified MRSA infection (case-patient) was matched—by veterinary referral hospital and by date of admission—to 2 control dogs with MSSA (the dogs seen immediately before and after the dog with MRSA). Dogs merely colonized by MRSA or MSSA were excluded from analysis.

### Data Collection

To collect information, we used medical records of all case-patient and control dogs to answer a pretested, standardized questionnaire. Data were collected on signalment, medical and surgical history, infection, hospital duration, and clinical outcome. Signalment data included breed, age, and sex. Medical and surgical history was limited to a 90-day period before admission to the referral hospital and included antimicrobial drug treatment, hospitalization, and surgical procedure. MRSA or MSSA infection data comprised site of infection and procedures performed before onset of infection, such as surgery, endoscopy, colonoscopy, intravenous catheterization, and urinary catheterization. Clinical outcome data covered whether surgery was required because of the infection and whether the animal was discharged, was euthanized, or died.

Because of a large number of categories, we recategorized several variables. Breed was categorized according to size based on weight: small (1–10 kg), medium (>10–25 kg), or large (>25 kg). Age was categorized as young (<2 years), middle aged (>2–8 years), or old (>8 years). Site (where MRSA or MSSA was cultured) was categorized as skin, ear, urinary, skeletal, and other (abdominal fluid, thoracic fluid, blood, oral cavity, lymph node, vagina, transtracheal wash fluid, and milk). The number of days dogs were hospitalized was categorized as short (<2 days), medium (3–7 days), or long (>7 days). Finally, specific antimicrobial drugs were grouped according to classes, i.e., aminoglycoside, β-lactam, chloramphenicol, fluoroquinolone, lincosamide, nitroimidazole, and tetracycline. For analytical purposes, dogs were subcategorized into 4 groups: did not receive any antimicrobial drug in the previous 90 days, received that specific class of antimicrobial drug, received another class of antimicrobial drug, and unknown status.

### Statistical Analyses

All descriptive statistics, model building, and analyses were performed by using Stata 10.0 (StataCorp, College Station, TX, USA) and by using exact logistic regression. In Stata, the score method was used for calculating p values, and the group option was applied to account for matching (*16*). All tests were 2 sided, and significance was based on p<0.05. For predictor variables, odds ratios (ORs) and 95% confidence intervals (CIs) were calculated. For descriptive variables listed in the categories of signalment, medical and surgical history, and infection, the outcome was defined as having MRSA or MSSA infection. Variables in the clinical outcome category were modeled as dependent variables, and MRSA or MSSA infection was the independent variable. To avoid problems associated with colinearity, we performed a correlation analysis to identify pairs of predictor variables that had high colinearity (|r|>0.8). We did not construct a multivariable model because of the relatively small sample size, resultant concerns of stability, and problems associated with overfitting. Consequently, we constructed only univariable models.

## Results

A total of 40 MRSA case-patients and 80 MSSA controls were eligible for inclusion. From each hospital, the number of case-patients and controls were, respectively, 7 and 14 (Ontario Veterinary College), 20 and 40 (Matthew J. Ryan Veterinary Hospital), and 13 and 26 (Angell Animal Medical Center).

Breed distribution was categorized according to weight (Table 1). Ages of MRSA case-patients ranged from 1 to 13 years (mean 5.6 and median 5.0 years). Ages of MSSA control dogs ranged from 6 months to 16 years (mean 6.8 and median 7.0 years). No distinction was made between intact or sterilized dogs. Overall, no significant differences appeared between case-patients and controls with respect to breed (p = 0.18), age (p = 0.50), or sex (p = 0.29).

**Table 1:** Univariable analysis of demographic (signalment) risk factors for MRSA versus MSSA infections in dogs, United States and Canada, 2001–2007*

Variable	MRSA, no. (%) dogs, n = 40	MSSA, no. (%) dogs, n = 80†	Odds ratio (95% CI)	p value‡
Breed, kg				
Small, 1–10	10 (25)	11/79 (13.9)	Ref	
Medium, >10–25	16 (40)	28/79 (35.4)	0.63 (0.19–2.01)	0.43
Large, >25	14 (35)	40/79 (50.6)	0.39 (0.12–1.25)	0.10
Age group, y				
<2	10 (25)	13 (16.3)	Ref	
3–8	20 (50)	43 (53.8)	0.63 (0.22–1.78)	0.34
>8	10 (25)	24 (30)	0.54 (0.15–1.91)	0.39
Sex				
F	14 (35)	36 (45)	Ref	
M	26 (65)	44 (55)	1.47 (0.65–3.45)	0.35

*MRSA, methicillin-resistant *Staphylococcus aureus*; MSSA, methicillin-susceptible *Staphylococcus aureus*; CI, confidence interval; Ref, referent category. Dogs with MRSA (case-patients) and MSSA (controls) infections were matched for veterinary referral hospital and date of admission. †Except as indicated. ‡Score method for estimating p values does not assume a symmetrical distribution for discrete data. p<0.05 was considered significant.

Regarding previous hospitalization or surgical procedures, no overall significant differences appeared between the MRSA and MSSA groups (p = 0.62 and 0.40, respectively). Results from the univariable analysis (Table 2) indicate that receipt of antimicrobial drugs (OR 3.84, 95% CI 1.21–14.74, p = 0.02), β-lactams (OR 3.58, 95% CI 1.04–14.79, p = 0.04), or fluoroquinolones (OR 4.61, 95% CI 1.08–27.37, p = 0.02), within 90 days before admission was significantly associated with a MRSA infection. Furthermore, when fluoroquinolones and β-lactams were included in the “other classes” category, the odds of a dog having MRSA versus MSSA infection increased over odds for dogs that had not received antimicrobial drugs (Table 2).

**Table 2:** Univariable analysis of medical and surgical risk factors for MRSA versus MSSA infections in dogs, United States and Canada, 2001–2007*

Variable†	MRSA, no. (%) dogs, n = 40	MSSA, no. (%) dogs, n = 80	Odds ratio (95% CI)	p value‡
Received antimicrobial drugs				
No	8 (20.0)	30 (37.5)	Ref	
Yes	26 (65.0)	33 (41.3)	3.84 (1.21–14.74)	0.02
Don’t know	6 (15.0)	17 (21.2)	1.29 (0.28–5.65)	0.75
Received >2 antimicrobial drugs				
No	25 (62.5)	56 (70.0)	Ref	
Yes	9 (22.5)	7 (8.8)	2.87 (0.81–11.49)	0.08
Don’t know	6 (15.0)	17 (21.2)	0.79 (0.19–2.79)	0.78
Received an aminoglycoside				
No	8 (20.0)	30 (37.5)	Ref	
Yes	1 (2.5)	1 (1.3)	5.71 (0.06–517.45)	0.30
Other classes§	25 (62.5)	32 (40.0)	3.80 (1.19–14.62)	0.02
Don’t know	6 (15.0)	17 (21.2)	1.29 (0.28–5.62)	0.75
Received a β-lactam				
No	8 (20.0)	30 (37.5)	Ref	
Yes	18 (45.0)	25 (31.3)	3.58 (1.04–14.79)	0.04
Other classes	8 (20.0)	8 (10.0)	4.18 (0.96–20.88)	0.04
Don’t know	6 (15.0)	17 (21.2)	1.31 (0.28–5.69)	0.75
Received chloramphenicol				
No	8 (20.0)	30 (37.5)	Ref	
Yes	1 (2.5)	2 (2.5)	2.61 (0.04–65.5)	1.00
Other classes	25 (62.5)	31 (38.8)	3.84 (1.21–14.74)	0.02
Don’t know	6 (15)	17 (21.2)	1.29 (0.28–5.65)	0.75
Received a fluoroquinolone				
No	8 (20.0)	30 (37.5)	Ref	
Yes	9 (22.5)	7 (8.8)	5.34 (1.24–27.38)	0.01
Other classes	17 (42.5)	26 (32.5)	3.24 (0.94–13.2)	0.06
Don’t know	6 (15.0)	17 (21.2)	1.31 (0.29–5.74)	0.75
Received a lincosamide				
No	8 (20.0)	30 (37.5)	Ref	
Yes	2 (5.0)	2 (2.5)	4.43 (0.27–75.86)	0.19
Other classes	24 (60.0)	31 (38.8)	3.77 (1.18–14.53)	0.02
Don’t know	6 (15.0)	17 (21.2)	1.32 (0.29–5.73)	0.75
Received a nitroimidazole				
No	8 (20.0)	30 (37.5)	Ref	
Yes	2 (5.0)	0	7.18 (0.53–∞)	0.07
Other classes	24 (60.0)	33 (41.3)	3.47 (1.08–13.33)	0.03
Don’t know	6 (15.0)	17 (21.2)	1.38 (0.29–6.17)	0.74
Received a tetracycline				
No	8 (20.0)	30 (37.5)	Ref	
Yes	2 (5.0)	1 (1.3)	6.63 (0.29–463.75)	0.17
Other classes	24 (60.0)	32 (40.0)	3.54 (1.11–13.65)	0.03
Don’t know	6 (15.0)	17 (21.2)	1.33 (0.29–5.88)	0.74
Hospitalized				
No	16 (40.0)	38 (47.5)	Ref	
Yes	16 (40.0)	25 (31.3)	1.54 (0.58–4.14)	0.37
Don’t know	8 (20.0)	17 (21.3)	1.17 (0.32–4.06)	0.08
Underwent surgical procedure				
No	25 (62.5)	46 (57.5)	Ref	
Yes	10 (25.0)	16 (20.0)	1.07 (0.39–2.83)	1.00
Don’t know	5 (12.5)	18 (22.5)	0.45 (0.09–1.68)	0.26

*MRSA, methicillin-resistant *Staphylococcus aureus*; MSSA, methicillin-susceptible *Staphylococcus aureus*; CI, confidence interval; Ref, referent category. Dogs with MRSA (case-patients) and MSSA (controls) infections were matched for veterinary referral hospital and date of admission. †Information obtained refers to the 90 days before admission to the veterinary referral hospital. ‡Score method for estimating p values does not assume a symmetrical distribution for discrete data. p<0.05 was considered significant. §Refers to dogs given antimicrobial drugs from other drug classes.

Most MRSA and MSSA infections were located on the skin. Overall, with regard to infection site, we found no significant difference between the MRSA and MSSA groups (p = 0.50) (Table 3). Before onset of the MRSA and MSSA infections, the most common procedure was intravenous catheterization—a significant risk factor for a MRSA infection (OR 3.27, 95% CI 1.14–10.65, p = 0.02). Neither colonoscopy nor endoscopy was performed on any animal. Dogs with MRSA infection were hospitalized 0–29 days (mean 3.4 and median 1.5 days), whereas dogs with MSSA infection were hospitalized 0–13 days (mean 2.0 and median 0 days). Overall, in terms of duration of hospitalization, we found no significant difference between case-patients and controls (p = 0.49).

**Table 3:** Univariable analysis of infection site, duration of hospitalization, and medical and surgical risk factors for MRSA versus MSSA infections in dogs, United States and Canada, 2001–2007*

Variable	MRSA, no. (%) dogs, n = 40	MSSA, no. (%) dogs, n = 80†	Odds ratio (95% CI)	p value‡
Site of infection				
Skin	19 (47.5)	38/78 (48.7)	Ref	
Ear	5 (12.5)	11/78 (14.1)	0.89 (0.21–3.28)	1.00
Skeletal§	7 (17.5)	6/78 (7.7)	2.69 (0.53–17.96)	0.23
Urinary¶	3 (7.5)	11/78 (14.1)	0.37 (0.03–2.20)	0.29
Other#	6 (15.0)	12/78 (15.4)	1.06 (0.24–4.14)	1.00
Duration of hospitalization				
Short (<2 d)	25 (62.5)	57 (71.3)	Ref	
Medium (3–7 d)	11 (27.5)	19 (23.8)	1.59 (0.52–4.94)	0.45
Long (>7 d)	4 (10.0)	4 (5.0)	2.70 (0.43–17.49)	0.23
Intravenous catheterization**				
No	21 (52.5)	58 (72.5)	Ref	
Yes	19 (47.5)	22 (27.5)	3.27 (1.14–10.65)	0.02
Surgery**				
No	25 (62.5)	58 (72.5)	Ref	
Yes	15 (37.5)	22 (27.5)	1.61 (0.65–4.09)	0.29
Urinary catheterization**				
No	36 (90.0)	77 (96.3)	Ref	
Yes	4 (10.0)	3 (3.8)	6.00 (0.48–314.98)	0.11

*MRSA, methicillin-resistant *Staphylococcus aureus*; MSSA, methicillin-susceptible *Staphylococcus aureus*; CI, confidence interval; Ref, referent category. Dogs with MRSA (case-patients) and MSSA (controls) infections were matched for veterinary referral hospital and date of admission. †Except as indicated. ‡Score method for estimating p values does not assume a symmetrical distribution for discrete data. p<0.05 was considered significant. §Specimens were from internal joint surface, joint fluid, intramedullary pin, and orthopedic implant. ¶Specimens were urine, urinary calculus, urinary catheter, and the wall of the urinary bladder. #Specimens were abdominal and thoracic fluids, blood, oral cavity swabs, lymph nodes, vaginal swabs, transtracheal wash fluid, and milk. **Procedures performed before infection occurred.

Surgery was required for treatment of 16 (40.0%) of 40 dogs with MRSA infection and 34 (42.5%) of 80 dogs with MSSA infection. Most dogs with MRSA and MSSA infections were discharged from the hospital (Table 4). For all dogs in the MRSA and MSSA groups that were euthanized, the infection was reported as the attributed cause of death. Overall, no significant differences were noted between case-patients and controls with regard to surgery (p = 0.79) or outcome (p = 0.64).

**Table 4:** Clinical outcome characteristics for dogs with MRSA and MSSA infections, United States and Canada, 2001–2007*

Variable	MRSA, no. (%) dogs, n = 40	MSSA, no. (%) dogs, n = 80†	Odds ratio (95% CI)	p value
Surgery required because of infection				
No	24 (60)	46 (57.5)	Ref	
Yes	16 (40)	34 (42.5)	0.89 (0.37–2.12)	0.84
Outcome				
Discharged	36/39 (92.3)	69/77 (89.6)	Ref	
Euthanized	3/39 (7.7)	8/77 (10.4)	0.63 (0.06–4.10)	0.71

*MRSA, methicillin-resistant *Staphylococcus aureus*; MSSA, methicillin-susceptible *Staphylococcus aureus*; CI, confidence interval; Ref, referent category. Dogs with MRSA (case-patients) and MSSA (controls) infections were matched for veterinary referral hospital and date of admission.

## Discussion

The identification of receipt of antimicrobial drugs—specifically β-lactams and fluoroquinolones—as risk factors for a MRSA infection was not unexpected. Data from human medicine and a logical hypothesis each indicate that antimicrobial drug use in animals would increase the likelihood of selection for multidrug-resistant bacteria such as MRSA. The case and control dogs included in this study were from veterinary referral hospitals; that is, tertiary care facilities that manage complicated medical and surgical cases referred from other veterinary facilities where treatment, surgery, or both might have been initiated. This study identified the highest prevalence of MRSA and MSSA infections from the skin (pyoderma) and ears (otitis), which in dogs are frequently treated with β-lactams and fluoroquinolones, respectively (*17*). Moreover, these conditions can become chronic and can result in repeated or prolonged antimicrobial drug treatments that might select for the development of antimicrobial drug resistance (*18*). Before admission to the referral hospitals, >50% of dogs with MRSA infection were given antimicrobial drugs from the β-lactam family. Methicillin resistance in staphylococci involves the *mecA* gene, which encodes for the penicillin-binding protein 2a and results in reduced affinity for all β-lactam antimicrobial drugs. Thus, medical management of MRSA cases can become complicated and can result in the administration of various classes of antimicrobial drugs (some of which can be ineffective), especially when culture and susceptibility testing have not been conducted.

In small animal medicine, fluoroquinolones are commonly used because of their activity against a wide range of bacteria and their ability to be given orally (*19*). In humans, administration of antimicrobial drugs, including macrolides (*7*), β-lactams (*20*), and fluoroquinolones (*7*,*21*), has been associated with increased risk for development of nosocomial MRSA infections compared with nosocomial MSSA infections. Specifically, use of fluoroquinolones has been positively correlated with the incidence of hospital-associated MRSA (*22*) infections. In addition to the direct effect of antimicrobial drugs on selection for antimicrobial drug–resistant organisms, other mechanisms could facilitate emergence of MRSA during fluoroquinolone treatment. Research performed by Bisognano et al. (*23*) demonstrated that fluoroquinolone-resistant MRSA and MSSA isolates exposed to subinhibitory levels of ciprofloxacin resulted in increased production of binding proteins, leading to higher levels of bacterial attachment. Thus, exposure to fluoroquinolones might promote the attachment of *S. aureus* while eradicating MSSA strains and might therefore promote acquisition of MRSA strains (*24*).

In our study, information pertaining to antimicrobial drug exposure in the 90 days before admission to the veterinary referral hospital was selected because that period was sufficient for adequate review of medical charts. In the literature, periods for antimicrobial drug exposure as a risk factor for MRSA acquisition have ranged from 1 to 12 months (*25*). Future studies with larger datasets might investigate the effect of varying time frames with respect to antimicrobial drug administration.

In dogs, identification of intravenous catheterization as a risk factor for MRSA infection was not unexpected. Intravenous catheterization has been associated with increased rates of MRSA infections in humans (*26*) and has been significantly associated with death of horses with MRSA infections (*27*). In previous studies as well as ours, however, intravenous catheterization might reflect a consequence of MRSA infection rather than a risk factor for development of MRSA infection.

Overall and with respect to outcome (discharged vs. euthanized), no significant differences between MRSA and MSSA infections were found. This finding is relevant for counseling clients, particularly considering the publicity regarding MRSA and the possible perception that MRSA infections are untreatable or carry a poor prognosis. Numerous studies in human medicine have compared mortality rates associated with MRSA and MSSA infections, but the results have been conflicting (*6*,*8*,*10*,*11*). Wang et al. (*6*) were unable to detect an association between higher mortality rates in patients with community-associated MRSA infections than in those with community-associated MSSA infections. Melzer et al. (*10*) were unable to demonstrate that mortality rates for patients with hospital-associated MRSA infections were significantly higher than those for patients with hospital-associated MSSA infections. Conversely, results from a retrospective cohort study conducted by Wang et al. (*8*), indicated that the mortality rate for patients with hospital-associated MRSA bacteremia was 1.78× higher than that for hospital-associated MSSA bacteremia.

In our study, inadequate epidemiologic definitions and veterinary surveillance data prevented us from classifying MRSA and MSSA infections as hospital or community associated. Nevertheless, other possible explanations as to why mortality rates between case-patients and controls did not differ significantly might in part be the predominant infection types and the retrospective aspect of the study. Most MRSA and MSSA infections were pyodermas and otitis externa or interna infections, which are superficial, rarely become invasive, and seldom result in death. Consequently, infection types for which death would be a more realistic possible outcome were limited, resulting in a corresponding limitation in statistical power. Comparison of mortality rates between patients with MRSA or MSSA infections would be best performed among only those with invasive infections and should be considered for future studies. Here, mortality rate information was obtained retrospectively and only recorded up to the time of discharge. Thus, whether dogs died from their infections after discharge from the referral hospital, causing an underestimate of deaths, is unknown.

Although our study was larger than previous studies, the power was still limited despite enrollment of 2 MSSA controls per each MRSA case-patient. Additional limitations were enrollment of case-patients and controls from referral hospitals and the use of matching. Because dogs in this study were from referral hospitals, extrapolation of results to the general dog population might be biased. In general veterinary practice, antimicrobial drug use, hospitalization, surgical procedures, and specific medical and surgical cases might differ considerably from those in referral hospitals. The incomplete medical records that accompanied case-patients and controls from referral hospitals might have affected responses to questions regarding previous medical or surgical procedures and antimicrobial drug use, all of which might have affected the results. Other potential risk factors such as underlying illnesses, admitting service, hospitalization locations (i.e., intensive care unit vs. hospitalization ward), and treatment cost were not investigated but could play a role in the development and outcome of MRSA infections in dogs. Finally, by using matching to control for potential confounders, the matched factors—date of admission and referral hospital—precluded the investigation of these variables as potential risk factors for a MRSA infection.

Despite these limitations, however, we found no identifiable differences between MRSA and MSSA infections in dogs with regard to signalment, types of infections, and clinical outcome. The prognosis for a dog with a MRSA infection is reasonably good. However, when determining that prognosis and when counseling owners, veterinarians should focus on the location and severity of infection rather than the bacterium involved. Furthermore, administration of β-lactams and fluoroquinolones were significant risk factors for the development of a MRSA infection. This finding strengthens the need for veterinarians to consider prudent antimicrobial drug–use guidelines and to restrict the use of fluoroquinolones as empirical or first-line therapy. Guidelines should recommend identification and susceptibility of the causal bacterial pathogen by performing a culture and susceptibility test. On the basis of susceptibility results, antimicrobial drugs should be dispensed at the proper dosage and duration for treatment and, in the absence of clinical disease, should not be prescribed.

Although only 4 risk factors were identified as being significantly associated with MRSA infection, results from the univariable analyses isolated several risk factors that have considerably large odds ratios or p values slightly greater than 0.05. With the exception of fluoroquinolones and β-lactams, measure of association for all other antimicrobial drug classes was reasonably higher for those dogs given specific antimicrobial drugs compared with those that were not. Because of the small sample size, however, the power of these associations was limited.

Our study shows that MRSA is an emerging pathogen in dogs, and risk factors for MRSA infection are similar to those identified in humans. Results from larger studies in the future might indicate that other classes of antimicrobial drugs, previous hospitalization and surgery, age, and the presence of a urinary catheter are also significantly associated with MRSA infections. Only larger sample sizes will provide more information on MRSA and MSSA infections and will determine more accurately other risk factors associated with MRSA infections in dogs.
